# Serum ferritin as a predictive biomarker for PEG IFNα-2b efficacy in chronic hepatitis B treatment

**DOI:** 10.3389/fcimb.2025.1603286

**Published:** 2025-09-08

**Authors:** Meiya Chen, Chenhao Wang, Miqiang Lin, Shiran Cai

**Affiliations:** ^1^ The Graduate School of Fujian Medical University, Fuzhou, China; ^2^ Department of Gastroenterology, The National Key Clinical Specialty, Zhongshan Hospital of Xiamen University, School of Medicine, Xiamen University, Xiamen, Fujian, China; ^3^ Department of Gastroenterology, The Second Affiliated Hospital of Fujian Medical University, Quanzhou, Fujian, China

**Keywords:** chronic hepatitis B, serum ferritin, HBsAg, PEG IFN-α2b, ALT

## Abstract

**Background:**

Chronic hepatitis B (CHB) caused by HBV infection leads to persistent liver inflammation. PEG IFNα-2b shows higher functional cure rates than oral antivirals, but identifying optimal candidates remains challenging. This study evaluates serum ferritin as a predictive biomarker for PEG IFNα-2b efficacy.

**Methods:**

Serum ferritin, HBsAg and transaminase were monitored in CHB patients treated with PEG IFN α-2b at baseline (0w), 12w and 24w. Patients were divided into the effective group (HBsAg reduction ≥1 log10 IU/mL or clearance) and the ineffective group (HBsAg reduction <1 log10 IU/mL) at 24 weeks. Similarly, for the results at 48 weeks, the patients were divided into the clearance group (achieving HBsAg clearance) and the non-clearance group (not achieving HBsAg clearance). To analyze the correlation between serum ferritin and the decrease of HBsAg, a multivariate logistic regression model was constructed. Potential confounding factors such as age, gender, HBeAg and HBV DNA status, ALT, AST, and iron metabolism indicators (FE, TIBC, TS) were included to evaluate the independent predictive effect of ferritin levels on the therapeutic effectiveness, calculate the corrected OR value and 95% CI. The predictive effect was evaluated by the ROC curve.

**Results:**

Among patients with chronic hepatitis B (CHB) who completed the PEG IFNα-2b course of treatment, the serum ferritin levels of all patients showed an increasing trend at baseline and during the treatment process, and the serum ferritin level in the effective treatment group (HBsAg reduction ≥1 log10 IU/mL or clearance) was significantly higher than that in the ineffective treatment group. It was confirmed by Spearman’s rank correlation analysis that the serum ferritin level was positively correlated with the decrease in HBsAg. Further analysis through the multivariate logistic regression model revealed that serum ferritin remained an independent predictor of the antiviral efficacy of PEG IFNα-2b. ROC curve analysis indicated that serum ferritin had a high predictive value for the antiviral efficacy of PEG IFNα-2b.

**Conclusion:**

The level of serum ferritin is closely related to the anti-hepatitis B virus efficacy of PEG IFNα-2b. It has great potential as a reliable and economical biomarker to guide treatment and optimize treatment strategies.

## Introduction

Hepatitis B virus (HBV) infection leading to chronic hepatitis B (CHB) is a major global health problem closely related to liver cirrhosis and liver cancer. This situation has imposed a heavy burden on health care systems around the world (Organization WH, 2025). Especially in low - and middle-income countries (LMICs), there are huge challenges in diagnosis and treatment. Expensive molecular tests (such as quantification of HBV DNA and HBV RNA), complex immunological analyses (such as quantification of anti-HBC), and tests that require professional equipment and personnel are difficult to be routinely carried out or unaffordable in many primary medical institutions. This has led to insufficient diagnosis, delayed treatment decision-making, and inadequate efficacy monitoring, ultimately limiting the optimized application of treatment strategies such as PEG IFNα and the benefit opportunities for patients. Achieving functional or clinical cure remains the ultimate therapeutic goal of CHB treatment (Wong et al., 2022). PEG IFNα is a first-line antiviral therapeutic drug, and its clinical cure rate is higher than that of oral antiviral drugs (Yeh et al., 2015; Song et al., 2021; He et al., 2022). However, individual responses to interferon therapy vary greatly, with some patients showing limited or no reduction in HBsAg levels.

Ferritin serves as the main protein for intracellular iron storage, predominantly localized in the liver, spleen, and bone marrow. It plays a significant role in liver inflammation and cellular damage. During liver inflammation or injury, ferritin functions as an acute-phase protein, released from hepatocytes into the bloodstream, resulting in a marked elevation in serum ferritin levels (Koperdanova and Cullis, 2015; Fonseca et al., 2023). Emerging research underscores the significance of serum ferritin in the pathophysiology of viral hepatitis (Yiannikourides and Latunde-Dada, 2019; Mancinelli et al., 2020). In the treatment of chronic hepatitis C, serum ferritin has been recognized as an independent predictor of interferon therapy outcomes (Lange et al., 2012). Studies have shown that ferritin may not only reflect liver inflammation but also play a role in forming antiviral immunity. Serum ferritin can regulate macrophage polarization (M1 and M2), and simultaneously affect the Th1/Th2 immune balance. It may promote the Th1-type cytokine environment conducive to viral clearance, thereby indirectly participating in antiviral immune regulation (Cronin et al., 2019). This is the key for PEG IFN-α2b to play its role. However, the relationship between serum ferritin and hepatitis B treatment remains poorly understood. To date, No studies have explored how serum ferritin levels change during PEG IFNα-2b treatment in chronic hepatitis B patients, nor their potential link to interferon efficacy.

Considering the close relationship between serum ferritin and hepatocyte function, this study aims to explore the changes in serum ferritin levels during the treatment of CHB patients with PEG IFNα-2b and its relationship with the treatment outcome. The aim is to determine a simple and reliable biomarker to assist clinicians in the early prediction and evaluation of interferon treatment outcomes, and ultimately optimize the treatment strategy for patients with chronic hepatitis B.

## Materials and methods

### Study participants

Data of patients who visited Zhongshan Hospital Affiliated to Xiamen University from October 2022 to October 2024 were retrospectively collected. All patients received monotherapy with nucleoside (acid) analogues (NAs) for ≥12 months before receiving PEG IFNα-2b treatment, but the treatment was ineffective. During the treatment with PEG IFNα-2b, NAs was continuously used to maintain virological inhibition. This study was approved by the Medical Ethics Committee of Zhongshan Hospital affiliated to Xiamen University (serial number 2024-021).

### Eligibility criteria

#### Inclusion criteria

Patients met the 2022 Guidelines for the Prevention and Treatment of Chronic Hepatitis B (You et al., 2023).

#### Exclusion criteria

- Patients with diagnoses of viral hepatitis types other than hepatitis B (e.g., hepatitis C or D), as well as alcoholic hepatitis, autoimmune liver disorders, cirrhosis, or hepatic cancer.
- Co-infection with HIV, EBV, or CMV.
- Presence of malignancies, hereditary/metabolic liver diseases, hematologic disorders, or severe renal dysfunction.
- A history of disorders related to iron metabolism.
- Pregnant or breastfeeding women and those with short-term fertility plans.
- Patients receiving iron supplementation or chelation therapy.

A total of 102 patients successfully finished the 24-week treatment period, while 59 patients completed the full 48 weeks. According to the Guidelines for the Prevention and treatment of Chronic Hepatitis B (2022 Edition), Outcome-based grouping was performed for analysis: patients at 24 weeks were classified as either effective (HBsAg reduction≥1 log10 IU/mL or clearance) or ineffective groups (HBsAg reduction<1 log10 IU/mL). Similarly, for the 48-week outcomes, patients were divided into clearance (achieving HBsAg clearance) and non-clearance groups (not achieving HBsAg clearance).

### Laboratory tests

In this study, patients received a standard antiviral protocol that combined 180 μg/week Peg-IFN α2b with nucleos(t)ide analogs. Serum samples were collected at three time points: before treatment (0w), at 12 weeks (12w), and at 24 weeks (24w). After fasting for 10 to 12 hours, 3-5ml of peripheral blood should be drawn at 7:00 - 9:00 in the morning and tested immediately after drawing. Blood specimens were collected using sterile dry tubes to measure hepatitis B markers, iron metabolism indicators (serum ferritin, serum iron, and total iron-binding capacity), ALT, and AST, or EDTA anticoagulation tubes for HBV DNA analysis. Serum or plasma was separated by centrifugation. Hepatitis B markers(HBsAg, Anti-HBs, HBeAg, Anti-HBe, Anti-HBc) and serum ferritin levels were determined by chemiluminescence immunoassay (CLIA), and HBV DNA was quantified by real-time fluorescence qPCR. The levels of serum iron and TIBC were determined by endpoint colorimetry, and the levels of ALT and AST were determined by continuous enzyme kinetics monitoring. Each method relies on automated equipment and is strictly controlled in quality to ensure accuracy.

Note: TS%=(Serum iron (FE, μmol/L)/Total iron binding capacity(TIBC, μmol/L))×100% .

### Statistical methods

The analysis was conducted using SPSS 25.0 version software. The normality of the data was evaluated through the Shapiro-Wilke test. Continuous variables that conform to the normal distribution are expressed as mean ± SD, and continuous variables that do not conform to the normal distribution are expressed as the median (IQR). Chi-square test was used for categorical data analysis. Independent t-test and Mann-Whitney U test were used for normal and non-normal continuous variables, respectively. For correlation analysis, Pearson’s method is applicable to normal data distributions, while Spearman’s method is applicable to skewed data sets. Based on the univariate analysis, a multivariate logistic regression model was further adopted to evaluate the independent predictive value of serum ferritin for the decrease of HBsAg. Potential confounding factors such as age, gender, HBeAg and HBV DNA status, ALT, AST, and iron metabolism indicators (FE, TIBC, TS) were included to construct the model. The final model was evaluated for the fitting effect by the Hosmer-Lemeshow goodness-of-fit test. And calculate the adjusted odds ratio (OR) and its 95%CI. The ROC analysis was used to obtain the optimal cut-off value and 95%CI, and to evaluate the predictive value of this index. When the AUC is greater than 0.5, this method is considered to have diagnostic value. A p value < 0.05 was considered statistically significant.

### Additional note

The degree of quantitative decline of HBsAg is used as the criterion for evaluating the antiviral efficacy.

This study used SPSSAU for therapeutic effect analysis. Retrospective efficacy analysis showed that the efficacy of serum ferritin (24w course of treatment, n=44 in the effective group, n=58 in the ineffective group) between the two groups with the existing sample size comparison was 84%, and the efficacy of 48w course of treatment, n=25 in the effective group, n=34 in the ineffective group) was 87.1%, indicating that this study could detect the difference at this level. Moreover, the risk of insufficient sample size affecting the results is relatively low. For details, please refer to **Table 1**.

**Table 1:** Retrospective efficacy analysis parameters and results.

Analyze the project	Parameter type	Parameters	24-week course of treatment	48-week course of treatment
Efficacy analysis	Inspection method		t-test	t-test
	α level		0.05	0.05
	Standard deviation(ng/mL) (Input)	Effective group (n1)	1436.7	795.1
		Ineffective groups (n2)	1084	744.1
	Sample size (input)	Effective group (n1)	44	25
		Ineffective groups (n2)	58	34
	Efficacy (Output)		0.84	0.87

The analysis was based on the actual observed sample size and standard deviation to evaluate the statistical power (efficacy) of detecting the differences in efficacy between groups of serum ferritin.

## Results

### Comparison of baseline and on-treatment characteristics between the effective and ineffective groups

**Table 2** shows the basic data characteristics of 102 patients with chronic hepatitis B (70 males and 32 females); The ratio of male to female was 2.2:1, and the average age was 41 ± 8 years old. There was no statistically significant difference in gender distribution and age between the two groups of patients (P>0.05). There was no statistically significant difference in the baseline negative rates of HBeAg and HBV DNA between the two groups of patients. After 24 weeks of treatment, a total of 25 people achieved HBV DNA clearance and 2 people achieved HBeAg serological conversion (both were patients in the ineffective group), but there was no significant statistical difference in the negative rate of HBV DNA and the negative rate of HBeAg between the two groups.

**Table 2:** The clinical data of patients with 24w treatment course were analyzed.

Items	Effective group (n=44)	Ineffective group (n=58)	P value
Male, n/%	34/77	36/62	0.155
Age (year)	40 ± 8	42 ± 8	0.207
HBV DNA neg, n/%	24/55	40/69	0.136
HBeAg neg, n/%	38/86	42/72	0.090
0w			
HBsAg (IU/ml)	60.69 (12.20,771.13)	604.26 (209.38,1051.00)	0.000
SF (ng/mL)	304.90 (156.53,533.78)	197.45 (69.63,409.90)	0.027
TS (%)	32.73 ± 16.06	34.46 ± 10.36	0.075
TIBC (μmol/L)	55.75 (53.00,60.23)	57.35 (52.70,62.70)	0.220
FE (μmol/L)	18.60 ± 8.19	19.59 ± 6.07	0.184
ALT (U/L)	30.80 (20.53,51.85)	22.65 (15.08,35.63)	0.021
AST (U/L)	26.05 (23.33,32.55)	23.05 (19.28,32.95)	0.022
12w			
HBsAg (IU/ml)	1.83 (0.13,44.75)	548.18 (97.19,1095.44)	0.000
SF (ng/mL)	1454.00 (977.95,2429.25)	706.00 (349.85,1397.25)	0.000
TS (%)	35.09 (29.28,42.89)	32.30 (26.90,43.12)	0.327
TIBC (μmol/L)	52.25 (48.70,57.58)	54.25 (50.58,58.43)	0.151
FE (μmol/L)	18.70 (16.10,22.25)	17.50 (15.05,22.80)	0.378
ALT (U/L)	77.45 (49.93,127.17)	59.80 (37.65,97.95)	0.060
AST (U/L)	65.75 (44.58,101.70)	54.79 (39.83,81.70)	0.226
24w			
HBsAg (IU/ml)	0.13 (0.00,2.52)	284.36 (68.98,788.47)	0.000
HBV DNA neg, n/%	39/89	50/86	0.716
HBeAg neg, n/%	38/86	44/76	0.186
SF (ng/mL)	1434.00 (898.43,2070.50)	684.10 (336.00,1252.25)	0.000
TS (%)	33.82 (27.76,44.03)	28.31 (24.44,36.31)	0.006
TIBC (μmol/L)	54.45 (49.70,57.28)	53.35 (51.25,60.23)	0.327
FE (μmol/L)	18.00 (15.98,23.60)	15.65 (13.25,20.35)	0.016
ALT (U/L)	73.00 (43.13,117.40)	49.30 (30.40,77.43)	0.037
AST (U/L)	65.30 (43.75,101.53)	47.60 (35.23,69.95)	0.036

Data are presented as mean ± standard deviation (SD) for normally distributed variables and median (interquartile range) for non-normally distributed variables.

HBsAg, hepatitis B surface antigen; HBeAg, hepatitis B e antigen; neg, negative; pos, positive; SF, serum ferritin; TIBC, total iron-binding capacity; TS, transferrin saturation; FE, serum iron; ALT, alanine aminotransferase; AST, aspartate aminotransferase.

At 0w, 12w, and 24w, patients in the effective group exhibited significantly lower HBsAg levels (P<0.05) and higher serum ferritin levels (P<0.05) compared to those in the non-effective group. Significant differences were observed between the two groups in ALT and AST levels at 0w, and in ALT, AST, TS, and FE levels at 24w, while no significant difference was found in TIBC.

### Comparison of baseline and on-treatment characteristics between the HBsAg clearance and non-clearance groups

Among the 59 participants who completed 48 weeks of treatment (**Table 3**), 42% (25/59) achieved HBsAg clearance. There were 47 male cases and 12 female cases, with an average age of 42 ± 9 years. There was no statistically significant difference in gender distribution and age between the HBsAg clearance rate group and the non-clearance rate group (P>0.05). At baseline, the negative rate of HBeAg in the HBsAg clearance group was significantly higher than that in the non-clearance group (P<0.05), while there was no significant difference in the negative rate of baseline HBV DNA between the two groups (P<0.05). Among these patients at 24w, a total of 23 achieved HBV DNA clearance and 1 achieved HBeAg serological conversion (as patients in the ineffective group). The negative rate of HBeAg in the HBsAg clearance group was still higher than that in the ineffective group (P<0.05). However, there was no significant statistical difference in the negative rate of HBV DNA between the two groups.

**Table 3:** The clinical data of patients with 48w treatment course were analyzed.

Items	HBsAg clearance (n=25)	HBsAg non-clearance (n=34)	P value
Male, n/%	21/84	26/76	0.478
Age (Year)	43 ± 8	41 ± 9	0.409
HBV DNA neg, n/%	12/48	20/59	0.410
HBeAg neg, n/%	25/100	26/76	0.016
0w			
HBsAg (IU/ml)	9.30 (0.65,52.09)	644.23 (97.10,1118.07)	0.000
SF (ng/mL)	335.60 (243.90,576.35)	244.95 (127.50,438.45)	0.037
TS (%)	36.54 ± 10.73	34.84 ± 13.19	0.599
TIBC (μmol/L)	55.80 (51.40,59.65)	57.35 (53.85,64.08)	0.099
FE (μmol/L)	19.94 ± 4.91	20.30 ± 7.62	0.826
ALT (U/L)	25.30 (20.10,37.75)	24.75 (15.08,33.45)	0.315
AST (U/L)	26.00 (22.55,30.90)	23.30 (20.03,29.93)	0.165
12w			
HBsAg (IU/ml)	0.12 (0.00,0.99)	294.67 (7.59,1083.49)	0.000
SF (ng/mL)	1285.00 (1122.00,2226.00)	914.50 (584.70,1548.75)	0.002
TS (%)	35.97 ± 10.18	35.19 ± 10.86	0.779
TIBC (μmol/L)	52.00 (47.95,56.75)	54.50 (50.48,57.85)	0.182
FE (μmol/L)	18.60 (14.35,21.30)	17.75 (15.38,21.73)	1.000
ALT (U/L)	61.90 (41.05,126.95)	60.35 (40.98,96.98)	0.679
AST (U/L)	61.70 (39.45,105.80)	58.55 (40.98,82.68)	0.226
24w			
HBsAg (IU/ml)	0.00 (0.00,0.00)	150.84 (1.05,640.33)	0.000
HBV DNA neg, n/%	25/100	30/88	0.130
HBeAg neg, n/%	25/100	27/79	0.017
SF (ng/mL)	1511.00 (1160.00,1919.00)	980.50 (479.75,1364.25)	0.001
TS (%)	32.73 (27.42,43.71)	31.22 (24.45,38.20)	0.407
TIBC (μmol/L)	53.80 (48.20,56.80)	55.00 (50.60,58.83)	0.192
FE (μmol/L)	17.90 (15.00,24.20)	17.25 (13.77,21.48)	0.550
ALT (U/L)	55.30 (40.70,87.75)	46.00 (29.95,69.93)	0.334
AST (U/L)	55.20 (39.60,86.20)	46.90 (34.95,69.50)	0.718

Serum HBsAg, serum ferritin, ALT, AST, TIBC, FE, and TS levels were assessed at baseline and during the course of treatment. The results showed that HBsAg levels were significantly lower in the clearance group than in the non-clearance group (P < 0.05), and serum ferritin levels were significantly higher in the clearance group (P < 0.05). However, there were no statistically significant differences between the two groups in ALT, AST, TIBC, FE, or TS levels (P > 0.05).

### Comparison of serum ferritin and ALT in chronic hepatitis B

**Figure 1:**
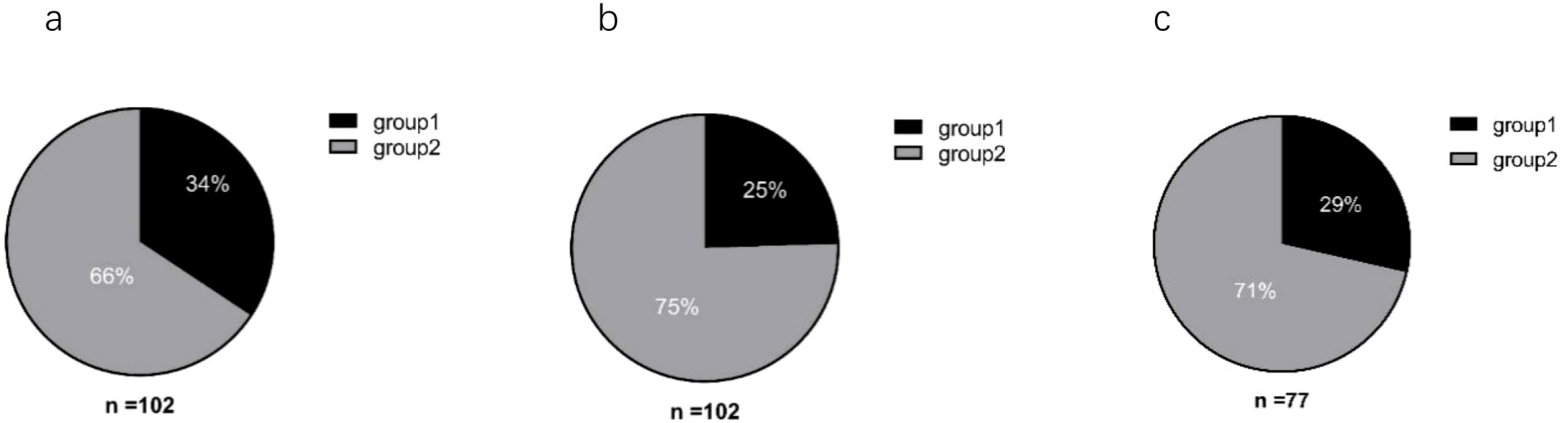
Analysis of baseline serum ferritin and ALT levels in patients. **(a)** Serum ferritin; **(b)** ALT. **(a, b)** Group 1: Baseline (0w) levels above the upper limit of normal (ULN); Group 2: Baseline (0w) levels within the normal range. **(c)** Group 1: Percentage of patients with normal ALT levels but serum ferritin above the ULN; Group 2: Patients with both ALT and serum ferritin within normal ranges.

Baseline (0w) serum ferritin and ALT values from the 102 patients were analyzed before initiating interferon therapy. Among these patients (**Figure 1**), 34% (35/102) had serum ferritin levels exceeding the upper limit of normal (Reference value range: 30–400 ng/mL for men, 13–150 ng/mL for women). In contrast, only 25% (25/102) exhibited ALT levels above the ULN (Reference value range: 7–40 U/L). Further analysis among patients with normal ALT values found that 29% (22/77) had serum ferritin levels exceeding the ULN.

### Statistical analysis of serum ferritin levels during treatment was performed

**Figure 2:**
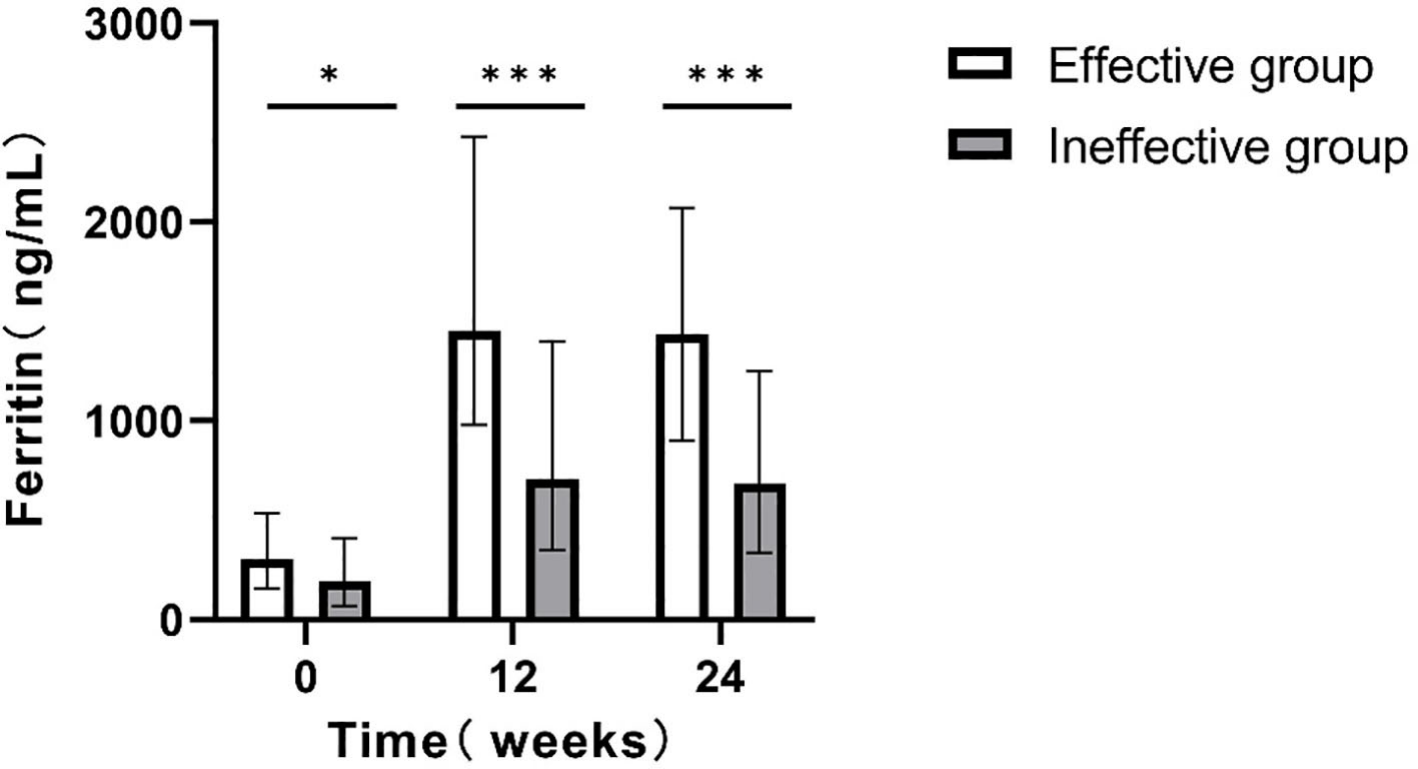
Comparison of serum ferritin levels at different time points between the two groups(24w). *p<0.05, **p<0.01, ***p<0.001.

Patients were first categorized based on their HBsAg reduction at 24 weeks into the effective group (HBsAg reduction ≥1 log10 IU/mL or HBsAg clearance) and the ineffective group (HBsAg reduction <1 log10 IU/mL). During PEG IFNα-2b treatment for 24w, serum ferritin levels in both groups were significantly higher than those before treatment (0w). Further comparison of the difference between the two groups showed that the serum ferritin level in the effective group was significantly higher than that in the ineffective group (P<0.05; **Figure 2**).

**Figure 3:**
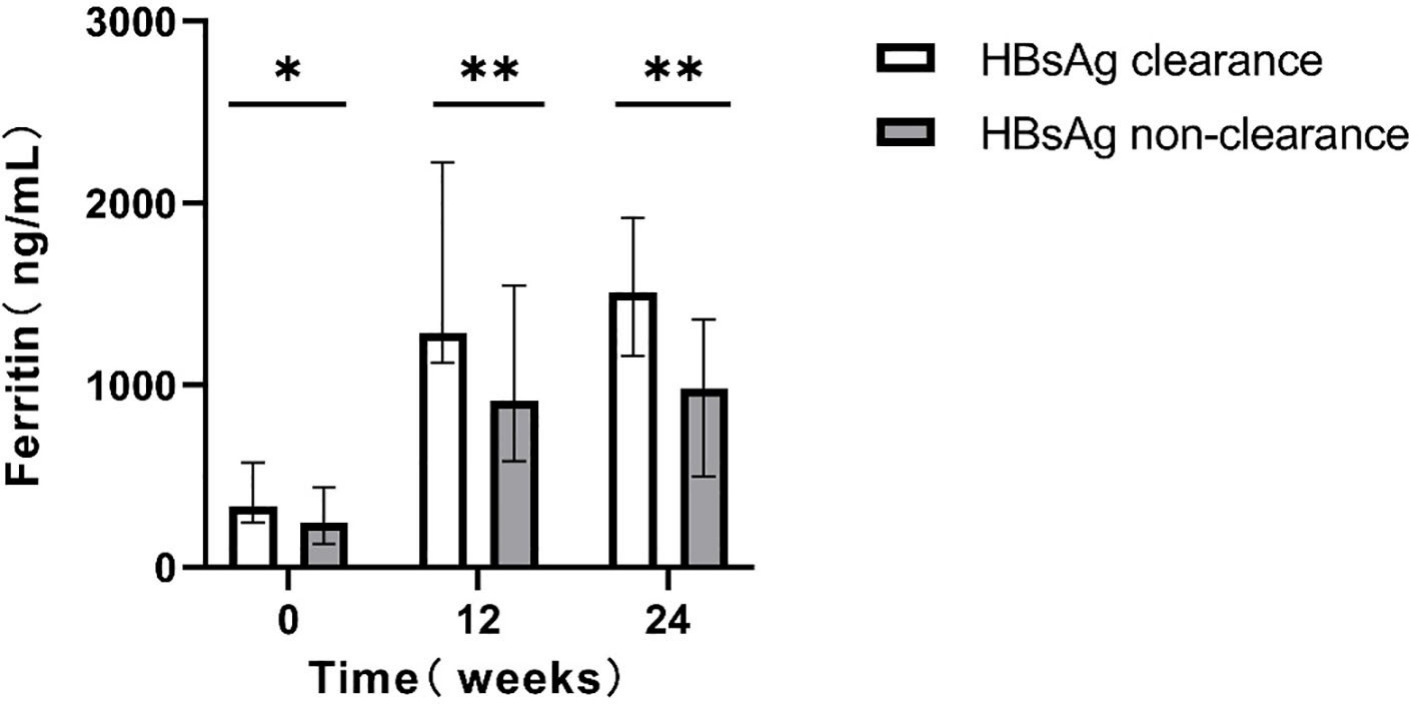
Comparison of serum ferritin levels at different time points between the two groups(48w). *p<0.05, **p<0.01, ***p<0.001.

For patients who completed the 48-week treatment course, intergroup comparisons were conducted. Patients achieving clearance exhibited significantly higher serum ferritin levels relative to those without clearance (P < 0.05; **Figure 3**).

### Correlation analysis between serum ferritin levels and HBsAg reduction in CHB patients treated with PEG IFN-α2b

**Figure 4:**
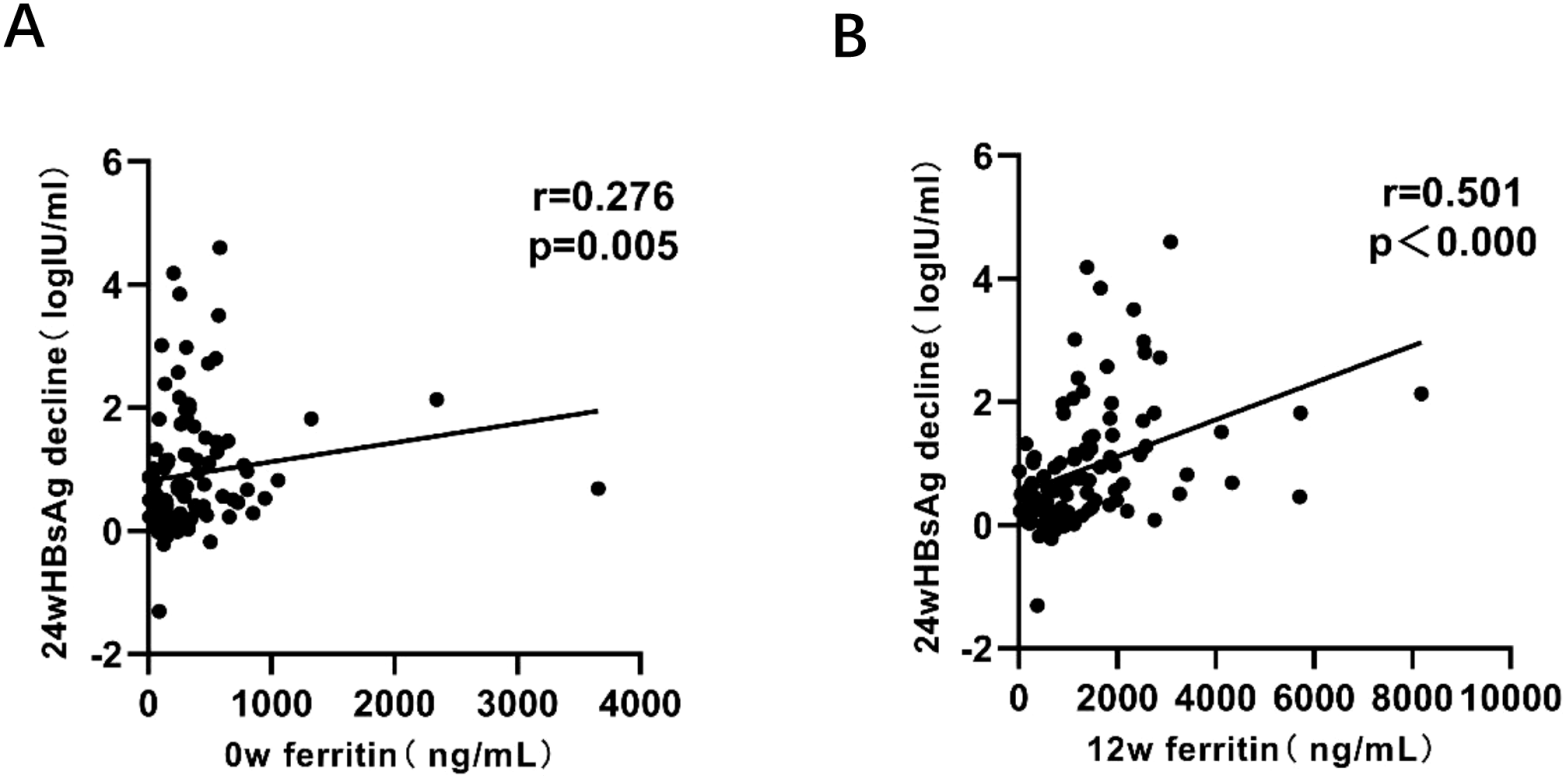
Correlation analysis between HBsAg reduction and serum ferritin levels at 0w, 12w. P < 0.05 indicates statistical significance. **(A)** Correlation between HBsAg reduction at 24w and serum ferritin levels at 0w. **(B)** Correlation between HBsAg reduction at 24w and serum ferritin levels at 12w.

The results revealed a significant positive relationship between HBsAg reduction at 24 weeks and serum ferritin measured at 0w, 12w. Notably, the strongest correlation was observed at 12 weeks (early stage of treatment), with a correlation coefficient of r=0.501, indicating statistical significance (**Figure 4**).

### Multivariate logistic regression verified the independent predictive efficacy of serum ferritin

We further evaluated the independent predictive value of serum ferritin levels at baseline (week 0), 12 weeks and 24 weeks of treatment for HBsAg decrease/clearance at 24 weeks and 48 weeks respectively by constructing a multivariate logistic regression model. The model included potential confounding factors such as age, gender, HBeAg and HBV DNA status, ALT, AST, and iron metabolism indicators (FE, TIBC, TS), and the stepwise regression method was used to screen the variables. The results indicated that 12w of serum ferritin had independent predictive power for the antiviral efficacy of 24w (OR=0.999, 95% CI=0.999-1.000, p=0.045) (**Table 4**.), and 24w of serum ferritin (OR=0.998, 95% CI=0.997-1.000, p=0.019) and 12w of serum ferritin (OR=0.999, 95% CI=0.997-1.000, p=0.048) had independent predictive power for the antiviral efficacy of 48w (**Table 5**).

**Table 4:** Multivariate logistic regression was used to verify the independent predictive efficacy of serum ferritin(24w).

Items	OR value	95% CI	P value
0w ferritin (ng/ml)	1.000	0.999,1.001	0.965
12w ferritin (ng/ml)	0.999	0.999,1.000	0.045

The included variables in the model were: age, gender, HBeAg status (positive/negative), HBV DNA status (positive/negative), ALT (U/L), AST (U/L), serum iron (FE, μmol/L), total iron binding capacity (TIBC, μmol/L), and transferrin saturation (TS, %). OR, odds ratio; CI, confidence interval. Group 1, effective group (HBsAg decrease ≥1log10IU/mL or HBsAg clearance; Group 2, Ineffective group (HBsAg Decrease<1log10IU/mL).

**Table 5:** Multivariate logistic regression was used to verify the independent predictive efficacy of serum ferritin(48w).

Items	OR value	95% CI	P value
0w ferritin (ng/ml)	0.998	0.995,1.001	0.238
12w ferritin (ng/ml)	0.999	0.997,1.000	0.048
24w ferritin (ng/ml)	0.998	0.997,1.000	0.019

The included variables in the model were: age, gender, HBeAg status (positive/negative), HBV DNA status (positive/negative), ALT (U/L), AST (U/L), serum iron (FE, μmol/L), total iron binding capacity (TIBC, μmol/L), and transferrin saturation (TS, %). OR, odds ratio; CI, confidence interval. Group 1, Clearance Group (HBsAg clearance); Group 2, non-clearance group (HBsAg not cleared).

### Predictive value of serum ferritin for the efficacy of PEG IFN-α2b in treating CHB

ROC curve analysis assessed the predictive value of serum ferritin levels at different time points for antiviral efficacy in CHB patients undergoing PEG IFNα-2b therapy. The area under the ROC curve (AUC) was calculated to determine the predictive ability of serum ferritin, using HBsAg reduction ≥1 log10 IU/mL or HBsAg clearance as the criteria for therapeutic efficacy.

**Figure 5:**
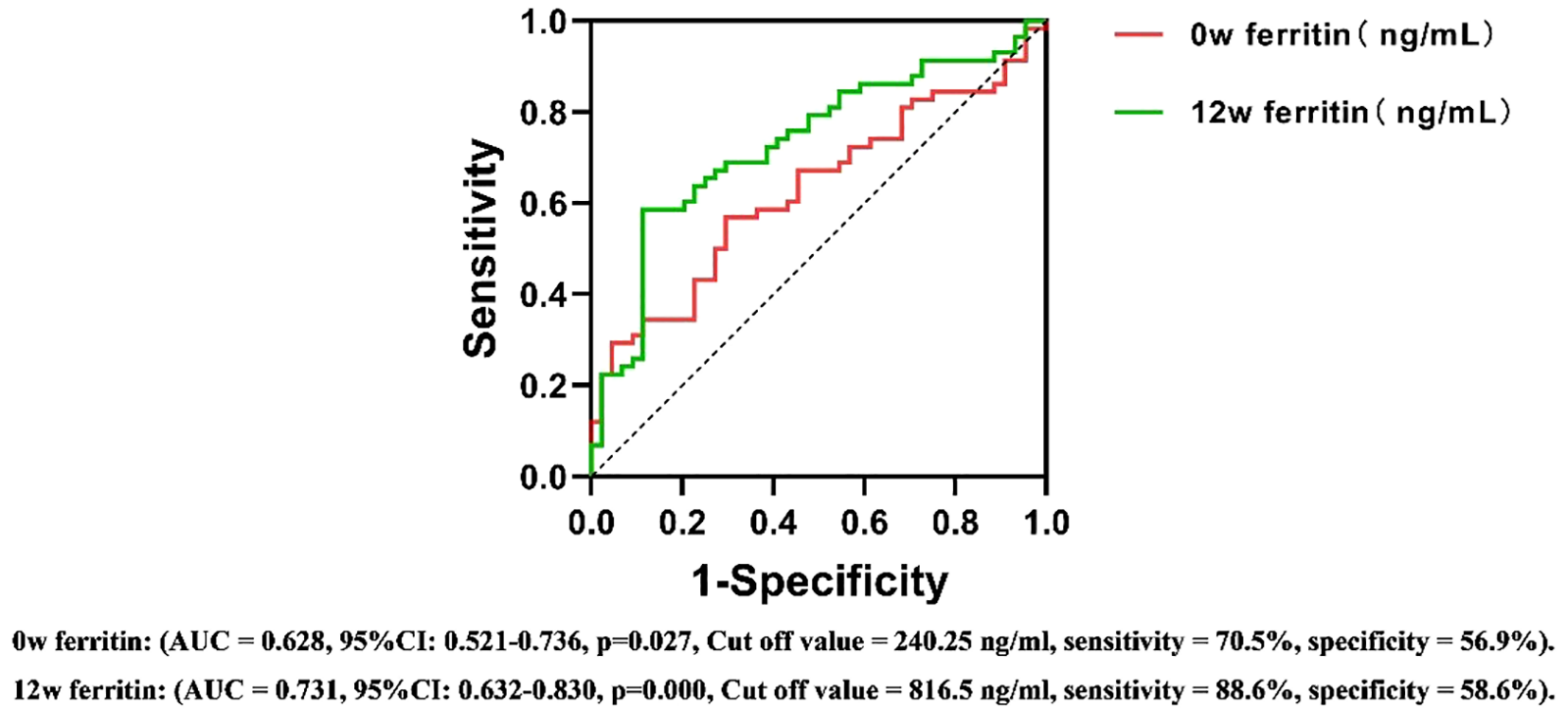
ROC curves for predicting the antiviral efficacy of PEG IFN-α2b in CHB patients based on serum ferritin levels at different time points(24w). AUC, Area Under Curve; CI, confidence interval.

**Figure 6:**
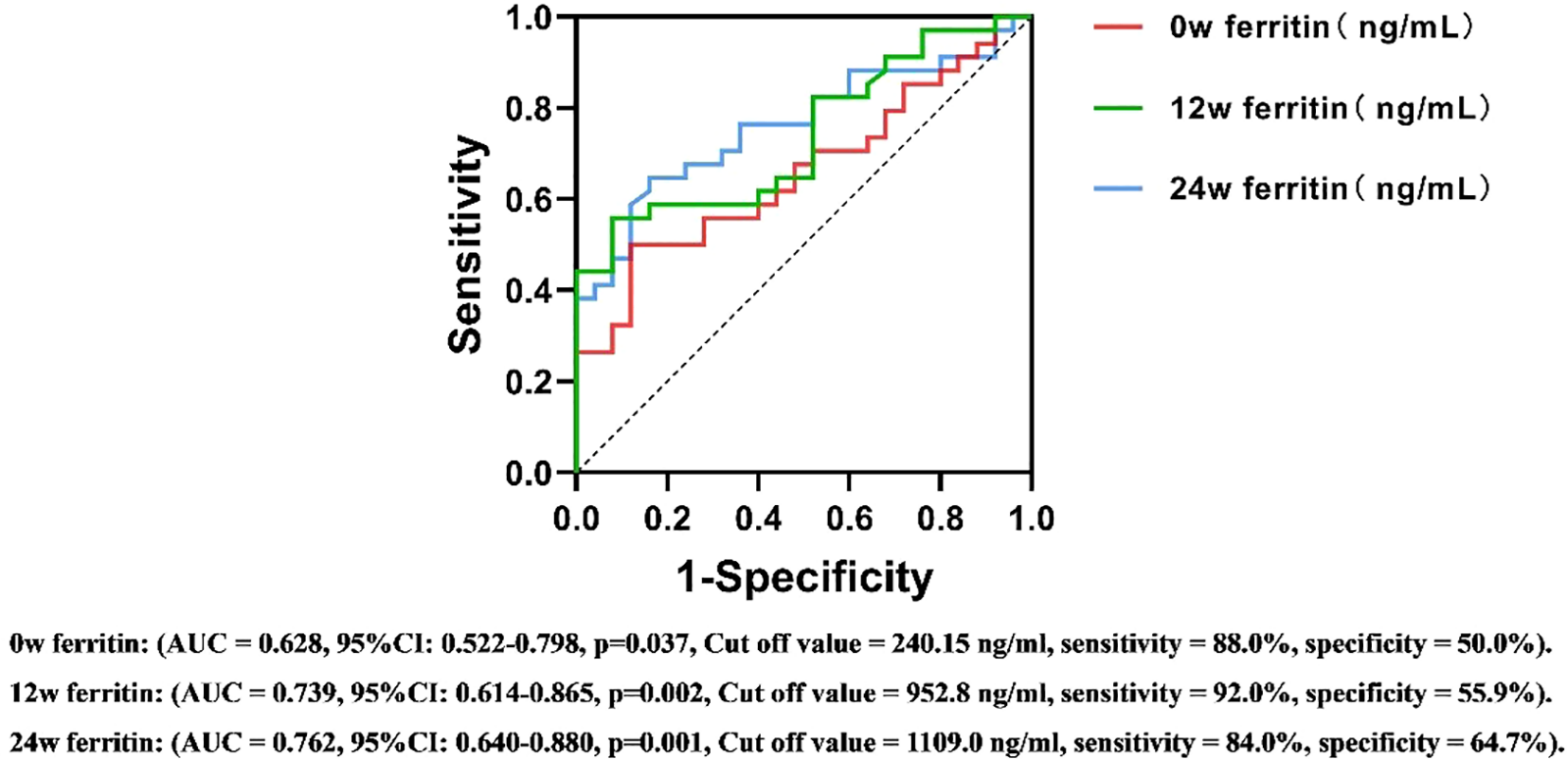
ROC curves for predicting the antiviral efficacy of PEG IFN-α2b in CHB patients based on serum ferritin levels at different time points(48w). AUC, Area Under Curve; CI, confidence interval.

For the 24-week outcomes, serum ferritin at 12w demonstrated the highest predictive performance (AUC = 0.731, Cut off value = 816.5 ng/ml, sensitivity = 88.6%, specificity = 58.6%, 95%CI: 0.632-0.830). For the 48-week outcomes, serum ferritin at 24w exhibited the strongest predictive value (AUC = 0.762, Cut off value = 1109.0 ng/ml, sensitivity = 84.0%, specificity = 64.7%, 95%CI: 0.640-0.880) (**Figures 5**, **6**). These results suggest that serum ferritin levels at 12w and 24w serve as reliable biomarkers for predicting antiviral treatment efficacy.

## Discussion

At present, PEG IFNα-2b is widely used in the clinical treatment of chronic hepatitis B (CHB), and its cure rate is significantly higher than that of oral antiviral drugs (Yeh et al., 2015). Its main advantage lies in the ability to eliminate Covalently closed circular DNA (cccDNA) in the nuclei of infected hepatocytes and integrate HBV DNA in chromatin, thereby achieving more comprehensive virus clearance (Wang et al., 2021). However, a considerable proportion of patients showed suboptimal or no response to this treatment, which was also accompanied by considerable side effects. Identifying early predictive biomarkers and selecting suitable candidates for PEG IFNα-2b therapy are now critical priorities in research. Existing biomarkers, such as HBcrAg, HBsAg-specific B cells, miR-548c-3p/TRIM22, and genetic phenotypes (Lin et al., 2023; Yin et al., 2023; Guan et al., 2024; Vecchi et al., 2024), have demonstrated potential but are seldom utilized in clinical practice. This highlights the urgent need for simple and widely applicable biomarkers to improve clinical outcomes.

This study provides evidence indicating that significant changes occurred in serum ferritin levels during the treatment of CHB with PEG IFNα-2b. Serum ferritin levels (measured at 0w, 12w and 24w) were positively correlated with the decrease of HBsAg at 24w, among which the correlation was the strongest at 12w. These findings established serum ferritin as a valuable early biomarker for predicting antiviral efficacy. To our knowledge, among CHB patients treated with PEG IFNα-2b, no previous studies have confirmed this relationship.

There are significant differences in fibrous inflammatory responses between acute hepatitis B (AHB) and chronic hepatitis B (CHB) infections, which are reflected in the dynamic changes of ferritin, HBsAg and ALT levels. In acute hepatitis B, a typical feature of infection is a strong immune-mediated inflammatory response against liver cells containing replicated HBV. This intense immune activation often leads to a significant increase in ALT, as liver cell damage is rapid and extensive during the acute phase. The HBsAg level in AHB usually peaks in the early stage of infection and decreases as the virus clearance proceeds. The conversion of serum to anti-HBs indicates that it has been resolved, which is consistent with the role of HBsAg as a marker of the active presence of the virus (Jindal et al., 2013). As an acute-phase protein, ferritin also increases in AHB, which is comparable to the severity of liver inflammation, especially significantly elevated in acute liver failure caused by acute hepatitis B. It can serve as a potential diagnostic and predictive marker for HBV-ACLF (Luo et al., 2023).

In contrast, our research focuses on chronic hepatitis B, where immune tolerance or partial immune activation may reduce the extent of ALT elevation. It is worth noting that some patients with chronic hepatitis B show elevated serum ferritin levels before treatment, even if the ALT level remains within the normal range. This finding emphasizes that ferritin is more sensitive as an inflammatory marker compared with ALT, especially in the early or mild liver disease (Agganis et al., 2018). Patients diagnosed with CHB who exhibit normal ALT levels may still experience marked hepatic inflammation and fibrotic changes (Zhang et al., 2021). During the interferon treatment process, the levels of serum ferritin and ALT in both groups of patients increased to varying degrees. However, the serum ferritin level in the effective group was significantly increased, while the ALT level showed no significant difference between the groups. This indicates that serum ferritin may be a more sensitive inflammatory biomarker than ALT. Furthermore, this difference may be partly attributed to the liver-protecting drugs used during the treatment.

Ferritin, as an acute-phase protein, is closely linked to liver inflammation and cellular damage. Elevated ferritin levels are indicative of more severe hepatic inflammation and fibrosis. Prior to treatment, effective responders exhibited lower HBsAg levels but higher ferritin levels compared to ineffective responders. Similarly, patients achieving HBsAg clearance at the end of treatment also displayed higher ferritin levels. These findings suggest that lower HBsAg levels may reflect greater hepatic inflammation in CHB patients, despite high HBsAg levels traditionally being considered predictive of poor outcomes (Cornberg et al., 2017). According to Wang et al., CHB patients who were HBeAg-positive with normal ALT levels exhibited a link between reduced HBV DNA levels and more severe liver fibrosis, potentially driven by HBV mutations and immune activation (Wang et al., 2024). Additionally, A correlation has been observed between PC mutations in the precore region of the HBsAg gene and lower HBsAg levels, as demonstrated in recent studies, which are correlated with increased inflammation and fibrosis (Lapalus et al., 2015; Kuhnhenn et al., 2018). Furthermore, the low HBsAg level of the patients may be related to the increased anti-HBV immune activity of the liver. In our study, we observed that although the HBsAg levels in the effective group were lower, the levels of ferritin, ALT and AST in these patients were higher, which contributed to achieving better therapeutic effects with interferon treatment.

Ferritin, responsible for intracellular iron storage, is essential for maintaining iron homeostasis and metabolism, and its levels are influenced by the body’s overall iron status (Zhao et al., 2024). Interestingly, other iron metabolism markers, such as serum iron, total iron-binding capacity, and transferrin saturation, showed no significant changes or differences between the groups during treatment. These findings indicate that elevated ferritin levels are primarily driven by immune and inflammatory responses instead of systemic changes in iron levels.

Current research indicates that inflammation and stress-related pathways significantly influence ferritin regulation. Under conditions of infection or inflammatory stimulation, macrophages exhibit increased ferritin release. In the HepG2 hepatocyte cell line, IL-1, IL-6, and TNF have been shown to induce ferritin synthesis (Torti and Torti, 2002). PEG IFNα-2b exerts its antiviral effects via immune modulation, specifically by activating key components of the innate immune system, such as NK cells, macrophages, and dendritic cells. It also induces the production of CXCL13 and IL-6, facilitating HBsAg clearance in CHB patients (Wang et al., 2021). Therefore, interferon exerts antiviral effects by regulating the immune response and indirectly promoting the synthesis and increase of serum ferritin levels. This is consistent with our research results. The serum ferritin levels of both groups of patients were significantly higher than the baseline levels, and the effective group was significantly higher than the ineffective group (**Figure 2**). Elevated ferritin levels can further feedback and enhance the activity of macrophages and T lymphocytes, improving their ability to recognize and clear virus-infected cells (Cohen et al., 2010; Cronin et al., 2019; Ni et al., 2022). Additionally, ferritin influences the function of NK cells (Ni et al., 2022), synergistically enhancing the antiviral effects of PEG IFN-α-2b by targeting and killing infected cells.

The stability of serum ferritin levels observed during the 12w to 24w treatment period may be attributed to receptor saturation and the subsequent activation of intracellular signaling pathways under a consistent dosage of PEG IFNα-2b. This phenomenon suggests that the initial treatment phase may have already achieved the maximum biological response, thereby limiting further changes in ferritin levels. While this hypothesis aligns with established concepts of immune and inflammatory pathway activation, further studies, including mechanistic investigations, are required to validate these findings and elucidate the underlying regulatory mechanisms. According to the results of multivariate logistic regression analysis, the serum ferritin level at 12 weeks has independent predictive efficacy for monitoring the treatment outcome at 24 weeks(OR=0.999, 95% CI=0.999-1.000, p=0.045), while the serum ferritin level at 24 weeks is a key independent factor for predicting the treatment outcome at 48 weeks (OR=0.998, 95% CI=0.997-1.000, p=0.019). After adding potential confounding factors such as age, gender, HBeAg and HBV DNA status, ALT, AST, and iron metabolism indicators, this conclusion remains robust. The ROC curve further evaluated its predictive value. At 12 weeks of treatment, early responders could be identified based on serum ferritin > 816.5 ng/ml, with a sensitivity of 88.6% and a specificity of 58.6%. Similarly, at 24 weeks of treatment, HBsAg clearance could be determined based on serum ferritin >1109 ng/ml, with a sensitivity of 84.0% and a specificity of 64.7%. This means that clinicians can more accurately assess the efficacy trend of PEG IFNα-2b antiviral treatment based on the dynamic changes of serum ferritin at 12 weeks and 24 weeks, and thus adjust the treatment strategy in a timely manner. This move not only helps to alleviate the related side effects caused by over-treatment, but also significantly reduces the unnecessary economic burden on patients, providing reliable biological markers and theoretical basis for the formulation of individualized treatment plans for chronic hepatitis B (CHB).

These findings provide valuable insights for clinical practice, emphasizing that serum ferritin is a simple and cost-effective biomarker for predicting the treatment outcome of PEG IFNα-2b in patients with chronic hepatitis B. Monitoring ferritin levels can enable timely adjustment of treatment strategies, thereby improving the success rate of treatment. Although these results are promising, further research is needed to explore their universality and biological basis.

This study has several notable limitations that should be emphasized. Firstly, as a retrospective analysis, this study may have inherent biases in patient selection, especially due to the lack of clinical data on patients with early CHB complicated with liver cirrhosis, which may limit the universality of the results. Secondly, the lack of adjustment for liver fibrosis, steatosis and metabolic parameters (such as insulin resistance and BMI) is a key limitation, as these factors independently regulate serum ferritin levels, which may affect the observed correlations. Finally, the relatively small sample size and the lack of subgroup analysis based on the HBeAg state may have affected the robustness of the conclusion. To confirm these findings and clarify the mechanism between ferritin dynamics and antiviral efficacy, large-scale multicenter studies are needed.

## Conclusion

This study confirmed that during the treatment of chronic hepatitis B patients with PEG IFN-α2b, the changes in serum ferritin levels were significantly correlated with the efficacy of interferon. Serum ferritin can be used as a potential predictive indicator for evaluating the therapeutic effect. As a low-cost and routinely accessible biomarker (with a detection cost much lower than that of molecular detection), the promotion and use of ferritin is expected to solve the treatment choice dilemma caused by the infeasibility of complex diagnoses in low - and middle-income countries (LMICs) with scarce resources. By identifying potential responders at an early stage, the clinical application of PEG IFNα-2b can be optimized (avoiding ineffective treatment and reducing medical expenditures), and the utilization efficiency of limited medical resources can be improved.

## Data Availability

The raw data supporting the conclusions of this article will be made available by the authors, without undue reservation.
